# Rhizodegradation of Petroleum Oily Sludge-contaminated Soil Using *Cajanus cajan* Increases the Diversity of Soil Microbial Community

**DOI:** 10.1038/s41598-020-60668-1

**Published:** 2020-03-05

**Authors:** Ibrahim Alkali Allamin, Mohd Izuan Effendi Halmi, Nur Adeela Yasid, Siti Aqlima Ahmad, Siti Rozaimah Sheikh Abdullah, Yunus Shukor

**Affiliations:** 1 Department of Biochemistry, Faculty of Biotechnology and Biomolecular Sciences, Universiti Putra Malaysia, 43400 UPM Serdang, Selangor, Malaysia; 2 Department of Microbiology, Faculty of Sciences, University of Maiduguri, P.M.B. 1069, Maiduguri, Borno State Nigeria; 3 Department of Land Management, Faculty of Agriculture, University Putra Malaysia, 43400 Serdang, Selangor, Malaysia; 4 Department of Chemical and Process Engineering, Faculty of Engineering and Built Environment, Universiti Kebangsaan Malaysia, UKM Bangi 43600, Selangor, Malaysia

**Keywords:** Biotechnology, Environmental biotechnology, Ecology

## Abstract

Most components of petroleum oily sludge (POS) are toxic, mutagenic and cancer-causing. Often bioremediation using microorganisms is hindered by the toxicity of POS. Under this circumstance, phytoremediation is the main option as it can overcome the toxicity of POS. *Cajanus cajan* a legume plant, was evaluated as a phyto-remediating agent for petroleum oily sludge-spiked soil. Culture dependent and independent methods were used to determine the rhizosphere microorganisms’ composition. Degradation rates were estimated gravimetrically. The population of total heterotrophic bacteria (THRB) was significantly higher in the uncontaminated soil compared to the contaminated rhizosphere soil with *C. cajan*, but the population of hydrocarbon-utilizing bacteria (HUB) was higher in the contaminated rhizosphere soil. The results show that for 1 to 3% oily sludge concentrations, an increase in microbial counts for all treatments from day 0 to 90 d was observed with the contaminated rhizosphere CR showing the highest significant increase (p  < 0.05) in microbial counts compared to other treatments. The metagenomic study focused on the POS of 3% (w/w) and based on the calculated bacterial community abundance indices showed an increase in the values for Ace, Cho, Shannon (Shannon-Weaver) and the Simpson’s (measured as InvSimpson) indices in CR3 compared to CN3. Both the Simpson’s and the Shannon values for CR3 were higher than CN3 indicating an increase in diversity upon the introduction of *C. cajan* into the contaminated soil. The PCoA plot revealed community-level differences between the contaminated non-rhizosphere control and contaminated rhizosphere microbiota. The PCoA differentiated the two treatments based on the presence or absence of plant. The composition and taxonomic analysis of microbiota-amplified sequences were categorized into eight phyla for the contaminated non-rhizosphere and ten phyla for the contaminated rhizosphere. The overall bacterial composition of the two treatments varied, as the distribution shows a similar variation between the two treatments in the phylum distribution. The percentage removal of total petroleum hydrocarbon (TPH) after 90 days of treatments with 1, 2, 3, 4, and 5% (w/w) of POS were 92, 90, 89, 68.3 and 47.3%, respectively, indicating removal inhibition at higher POS concentrations. As the search for more eco-friendly and sustainable remediating green plant continues, *C. cajan* shows great potential in reclaiming POS contaminated soil. Our findings will provide solutions to POS polluted soils and subsequent re-vegetation.

## Introduction

The global over-dependent on fossil fuels as sources of energy increases the rate and extent of exploration, transportation, refining and storage of crude oil by the petroleum industries^1,2^. Petroleum industries produce a large amount of leftover from petroleum oil as a residue, which is called petroleum oily sludge (POS). The indiscriminate disposal of inadequately treated oily sludge to the environment is causing a lot of environmental and human health problems. All environmental protection agencies and the experts have classified oily sludge as a hazardous organic waste. Oily sludge is a complex mixture of organic compounds and inorganic metalloids, of which the structure and bioavailability of these compounds resist natural degradation. POS constitutes 50–80% hydrocarbon, 10–30% solid material, 20–30% water and 5–10% heavy metals^3^. Several physical and chemical methods are being extensively used for the remediation of oily sludge from temporary deposit sites and contaminated environments. However, these methods tend to be expensive and environmentally unfriendly. An alternative is biological methods which can provide an effective and eco-friendly approach. By making use of these kinds of technologies, the content of harmful components is often decreased or eradicated, as well as its negative environmental and health influences can, therefore, be reduced^4,5^.

Nevertheless, because of the recalcitrant character of POS^6^, the quest for remediation strategies together with the requirement of fulfilling stringent environmental laws and lowering the cost of remediation is a continuing process^3^. The complexity of oily sludge composition of hydrocarbon, heavy metals and others made it hardly bioavailable for microbial degradation^5^. In Malaysia, POS waste falls under the scheduled waste with code SW 311, and about 4,293 tons were generated in 2015 alone (DOE, 2012).

Phytoremediation is one of the strategies of bioremediation, and it uses selected plants to cleanup polluted environment. Plants are more tolerant in general to excessive concentrations of POS compared to microorganisms^7^. Consequently, phytoremediation is potentially a fresh possibility of a much more effective and sustainable answer for the remediation of POS-contaminated soils^8^. Numerous plants have already been recognized to assist in the phytoremediation of areas polluted with petroleum hydrocarbons, and great number of research have identified grasses and legumes due to their reportedly greater tolerance to hydrocarbon and also due to their other beneficial qualities^9^. The plant- microorganisms connection has a tendency to boost the degradation of contaminants in the rhizosphere by way of a symbiotic connection. Plants can promote microbial activity anywhere from 10 to 100 times greater in the rhizosphere through the release of exudates that contain compounds including amino acids, carbohydrates and flavonoids^10^. The secretion of these nutrients-containing exudates offers nitrogen and carbon sources to soil microorganisms. In addition, this process generates an environment that is nutrient-rich in which the activity of microorganisms can be activated. Along with the capability to secrete compounds that can facilitate the activity and growth of rhizospheric microorganisms, plants can furthermore discharge specific enzymes that have the capability to degrade pollutants. The compounding effect results in a more efficient remediation of toxicants, including POS^11^.

Legumes are known to have the edge over non-leguminous plants in the process of phytoremediation due to their capacity for nitrogen fixation^12^ and therefore, do not need to contest with microorganisms as well as other plants for the constrained resources of accessible soil nitrogen. Common desirable characteristics of these plants are the ability to fix nitrogen (a major limiting factor for effective degradation of pollutants) which is strategic to establish nutrient-rich rhizosphere as it is known that areas contaminated with oily sludge have a deficit in the C:N ratio^9^. The legume-bacterial interaction is a synergistic strategy of which leguminous plants provide the microorganisms in the rhizosphere with growing space and essential nutrients, as the microorganisms use their degrading machinery to biodegrade hydrocarbon pollutant in their surroundings^13^. Additionally, rhizo- and endophytic microorganisms, in collaboration with the legume improve the supply of nutrient and growth hormones that can promote plants’ growth, and at the same time increase hydrocarbon bioavailability^14–17^. *Cajanus cajan* (pigeon pea) is also known as Kacang dhal in Malay. It is a very common legume crop in tropical countries. The plant provides an important source of protein to the diet of human. It features a lengthy root system which could endure in various soil types^18^. It can withstand the alterations in pH over a broad range. In addition, the plant is known to grow at a broad range of temperature from 10 to 35 °C^19^. In India, this plant is utilized as a multi-purpose plant in the agroforestry industry, in which its many functions include as sources of firewood, manure, food and fodder^19^. Although the plant species utilized in this study has been documented to degrade polluted soil contaminated with spent engine^20^, no prior research has documented its use in the remediation of POS-contaminated soil. In this study, we report for the first time, the phytoremediation of POS in soil using *Cajanus cajan*.

## Materials and Methods

### Experimental setup

The experiment was carried out in the Faculty of Biotechnology and Biomolecular Sciences, Universiti Putra Malaysia. The petroleum oily sludge (POS) used in this study was obtained from a Shell refinery center in Port Dickson, Negeri Sembilan Malaysia in 2014. The POS is in the form of clay mud and black in colour with a pH of 7.12^21^. The soil was collected from an agricultural farm in Universiti Putra Malaysia. The seeds of *C. cajan* (Pigeon pea) and plastic pots were obtained from a local grocery company.

## Experimental Design

### Pot preparation

**Figure 1:**
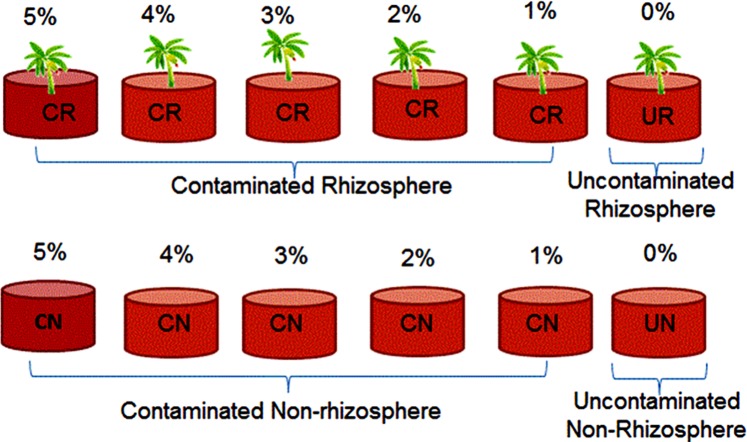
Experimental design showing the various treatments for POS-contaminated soil. The labels CR, CN, UR and UN represent contaminated rhizosphere, contaminated non-rhizosphere, uncontaminated rhizosphere and uncontaminated non-rhizosphere, respectively.

Soil, which has been previously air-dried and sieved using a 2 mm sieve was placed in each corresponding pot (3 kg), and labelled as contaminated rhizosphere (CR), contaminated non-rhizosphere (CN), uncontaminated rhizosphere (UR) and uncontaminated non-rhizosphere (UN) (Fig. 1).

### Treatment of soil with POS

POS was added into each pot to the final concentrations of 1%, 2%, 3%, 4%, and 5% (w/w) and homogenously mixed. The soil was properly agitated and thoroughly mixed to ensure homogeneity and uniformity of soil to the POS in a fume hood. The pots were allowed to remain undisturbed for one week for the volatilization processes. The soil sample was taken for physicochemical analysis before treatment. After one week, seeds of *C. cajan* were planted accordingly. Three independent experiments were carried out.

## Planting of Seeds

### Seed viability test and breaking of dormancy

The viability of the seed of *C. cajan* was tested using the floatation method. The seeds were tested for viability by soaking it in distilled water for 5 min. After that, all floating seeds are sieved out as non-viable and the water was drained immediately from the seeds that sank which are now considered viable seeds. The seeds were then surface sterilized with 10% hydrogen peroxide solution before planting. The seeds was sown to a depth recommended for *C. cajan*, which is from 1.5 to 3 cm^22^. For an excellent establishment of the plants, five seeds were planted in a hole. Likewise, the respective pots were irrigated every day and emergence was observed subsequently. The plants were moderately watered every two days with tap water. The appearance of the plants in response to the presence of POS in soil was monitored to determine if there is any phytotoxicity of POS to the plants.

### Microbiological analysis

The media used were Nutrient Agar (NA); for the enumeration and isolation of bacteria, mineral salts media and a modified Bushnell and Haas media the for isolation of hydrocarbon-utilizing bacteria referred to here as oil agar (OA) where diesel oil (0.1% w/v) was used as the carbon source in the medium^23^.

### Culture dependent microbial analysis

The bacterial population of the soil samples for 0, 30, 60, and 90 days was enumerated by serially diluting 1 g of soil sample collected from the rhizosphere of *C. cajan*. Suitably diluted samples (10^−5^, 10^−6^ and 10^−7^) were transferred into the already prepared media using a dropper pipette, 500 µL of each dilution was inoculated on NA and OA which contained 0.1% v/v diesel agar surface^23^. The plates were incubated at 30 °C for 24 h, for NA, and five days for OA, respectively^23^. The number of viable total heterotrophic rhizobacteria and hydrocarbon-utilizing bacteria in the soil samples was estimated from the number of colonies formed using a colony counter.

### Culture-independent microbial analysis

Culture-independent bacterial community in *C. cajan* rhizosphere after 90 days was determined using metagenomics analysis of the rhizospheric soil collected from the contaminated rhizosphere CR of 3% oily sludge and contaminated non-rhizosphere CN of 3% oily sludge as control.

### Soil extraction and PCR amplification

Microbial DNA was extracted from samples in treatments CN3 and CR3 after 90 days using Power soil^®^ DNA Isolation Kits (MOBIO, USA) following the manufacturer’s instruction. The marker region of the bacteria was amplified by PCR using a PCR (BioRad Thermal Cycler, United Kingdom) with the following conditions; 95 °C for 2 min, followed by 25 cycles at 95 °C for 30 s, 55 °C for 30 s, and 72 °C for 30 s, and a final extension for 5 min using the primers sequences 5'- barcode 2 - (reverse primer sequence) −3'and 5'-barcode 1- (forward primer sequence) – 3', where the barcode consists of an eight-base sequence that is sample-specific and distinctive to each sample. The PCR reaction was carried out in triplicate 20 µL-mixtures containing 2 µL of 2.5 mM dNTPs, 4 µL of 5 × FastPfu buffer, 0.4 µL of FastPfu Polymerase, 0.8 µL of each primer (5 µM) and 10 ng of template DNA. Extraction of amplicons was carried out from a 2% agarose gel. Purification of the amplicons was carried out utilizing AxyPrep DNA Gel Extraction Kit (Axygen Bioscience, Union City, CA, USA) in accordance to the instruction furnished by the manufacturer and quantified using QuantiFluor^TM^ –ST (Promega, USA). Construction of the library was carried out utilizing Linked ‘Y’ adapter; with adapter dimer removed by utilizing beads and the library was concentrated via PCR and hydroxides utilized in the generation of single-stranded DNA fragments. Sample libraries were then pooled in equimolar concentrations, and paired-end sequenced using the Illumina MiSeq platform (2 × 250/300 bp) according to standard protocols.

## Data analysis

Several criteria were utilized for demultiplexing raw Fasta files and QIIME-based quality-filtration (version 1.9.1). The criteria include overlapped relationship and merging of paired-reads into a single read. The merged reads were then utilized for the clustering of operational taxonomic units (OUT), classification of taxonomy and assessment of community diversity. The software Trimmomatic was utilized for processing sequence reads^24^, and then the reads were assembled utilizing the software Flash^25^ followed up by more analysis using the software MOTHUR v 1.33.0^26^. Alignment of unique sequences was based to the SILVA database with the settings put to default. In addition, removal of chimeric sequences was carried out. Obtained sequences that passed the screening process were then classified using the Ribosomal Database Project naïve Bayesian rRNA classifier at a confidence of 80%. The proportion of sequence identities was calculated at each taxonomic level as the percentage of all sequences classified in that particular sample. Classification of OTUs was based on the similarities of 97%. The software MOTHUR was utilized in the classification of the alpha-diversity indices, which include observed OTUs (Sobs), Ace, Chao, Shannon and InvSimpson indices. PCoA or principal coordinates analysis conducted in R (Version 3.1.2) was utilized to find the bacterial community structure, which is based on the OTU composition.

## Measurement of biodegradation in the soil

### Gravimetric method

Five grams of soil from all treatments were collected and transferred into 100 mL conical flasks. Then 50 mL of hexane was added and shaken on a Protech orbital shaker (USA) at 150 rpm for 24 h. The layer separating the solvent with oil and the soil was transferred to a pre-weighted clean conical flask, the sample was left overnight in a fume hood for the evaporation of the solvent, and the amount of residual TPH was gravimetrically determined using the formula.

$$ \% \,{\rm{Biodegradation}}=\frac{{\rm{Weight}}\,{\rm{of}}\,{\rm{oil}}\,({\rm{control}})\,-\,{\rm{Weight}}\,{\rm{of}}\,{\rm{oil}}\,({\rm{degraded}})}{{\rm{Weight}}\,{\rm{of}}\,{\rm{oil}}\,({\rm{control}})}\times 100$$

## Results and Discussion

### Total heterotrophic rhizosphere bacterial count

**Figure 2:**
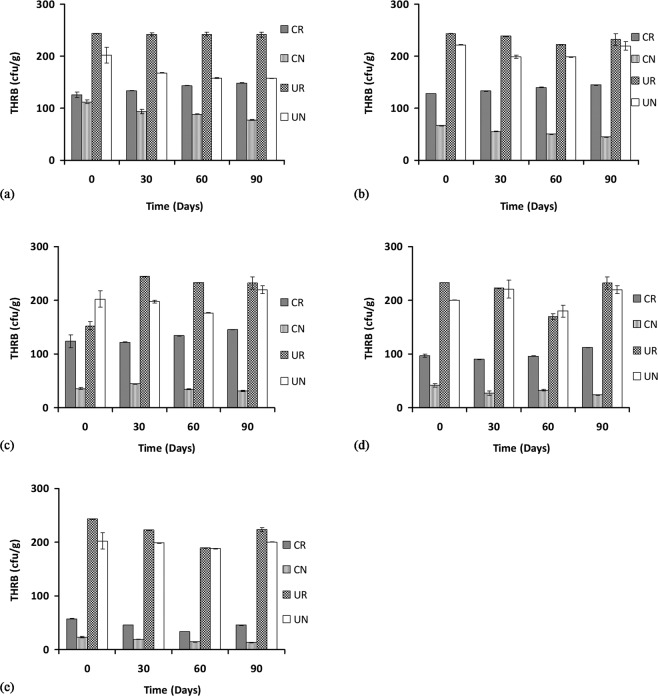
Total heterotrophic rhizospheric bacterial counts at 1, 2, 3, 4, and 5% concentration of petroleum oily sludge labelled (**a**), (**b**), (**c**), (**d**) and (**e**), respectively, under various treatments presented as mean ± standard deviation (n = 3). CR: Contaminated rhizosphere, CN: Contaminated non-rhizosphere, UR: Uncontaminated rhizosphere, UN: Uncontaminated non-rhizosphere.

The total counts for the heterotrophic rhizospheric bacteria (THRB) were enumerated at 0, 30, 60 and 90 days of treatments at various concentrations of POS. The result shows that for 1% POS, the THRB counts for contaminated rhizosphere or CR increased significantly (p < 0.05) but slightly from 125 × 10^7^ to 148 × 10^7^ CFU/g, while the count for contaminated non-rhizospheric CN decreased significantly (p  <  0.05) from 112 × 10^7^ to 77.3 × 10^7^ CFU/g, the counts for the uncontaminated rhizosphere UR stayed about the same from 243 × 10^7^ to 241 × 10^7^ CFU/g and the counts for the uncontaminated non-rhizosphere UN decreased significantly (p  <  0.05) from 202 × 10^7^ to 157 × 10^7^ CFU/g (Fig. 2(a)).

The THRB counts for the 2% POS concentration from 0 to 90 days show a significant (p < 0.05) increase in rhizospheric CR counts from 12.7 × 10^7^ to 144.8 × 10^7^ CFU/g, no significant change (p > 0.05) to the contaminated non-rhizospheric CN count (from 66.3 × 10^7^ to 44.7 × 10^7^ CFU/g), no significant change (p > 0.05) to the uncontaminated rhizospheric UR count (243 × 10^7^ to 232 × 10^7^) CFU/g and also no significant change (p > 0.05) to the uncontaminated non-rhizospheric UN count (from 221 × 10^7^ to 219 × 10^7^) CFU/g (Fig. 2(b)).

The THRB counts for the 3% POS concentration from 0 to 90 days show that the contaminated rhizospheric counts CR were increased but not significant (p > 0.05) (from 123 × 10^7^ to 145.4 × 10^7^ CFU/g), the contaminated non-rhizospheric CN counts were not increased significantly (p > 0.05) (from 35.8 × 10^7^ to 31.3 × 10^7^) CFU/g, the uncontaminated rhizospheric UR counts were increased significantly (p < 0.05) from 152 × 10^7^ to 232 × 10^7^ CFU/g, and the THRB counts in the uncontaminated non-rhizosphere UN were also not increased significantly (p > 0.05) (from 202 × 10^7^ to 219 × 10^7^) CFU/g (Fig. 2(c)).

The THRB counts for the 4% POS concentration from 0 to 90 days shows that the contaminated rhizospheric count CR was not significantly increased (from 96.7 × 10^7^ to 112 × 10^7^ CFU/g), the contaminated non-rhizospheric CN counts were decreased significantly (p < 0.05) (from 41.6 × 10^7^ to 23.7 × 10^7^ CFU/g), the uncontaminated rhizospheric UR counts were not significantly (p > 0.05) increased (from 233 × 10^7^ to 232 × 10^7^ CFU/g) and similarly in the uncontaminated non-rhizospheric UN, the bacterial counts were not significantly (p > 0.05) increased (from 200 × 10^7^ to 219 × 10^7^ CFU/g) (Fig. 2(d)). At the highest POS concentration tested (5%), there were a decreasing trend or no change in overall for the THRB counts. For example, for the contaminated rhizospheric CR counts, a decrease was observed (from 57.7 × 10^7^ to 45.4 × 10^7^ CFU/g), but this decrease was not significant (p > 0.05), in the contaminated non-rhizosphere CN counts, a reduction from 23 × 10^7^ to 13.2 × 10^7^ CFU/g was observed and this reduction was significant (p < 0.05). In the uncontaminated rhizospheric UR bacterial count, a reduction from 243 × 10^7^ to 223 × 10^7^ CFU/g was observed, but similarly, this reduction was not significant (p < 0.05) and finally, in the uncontaminated non-rhizospheric UN counts, there were no significant changes (p > 0.05) observed (from 202 × 10^7^ to 200 × 10^7^ CFU/g) (Fig. 2(e)).

In general, the results show a significant (p < 0.05) increase in the numbers of THRB counts in the uncontaminated rhizosphere (UR) compared to the uncontaminated non-rhizospheric (UN) bacterial counts. Although the microbial community in the rhizosphere is about 10–100 times higher than that of the non-rhizosphere^10^, the increase in the THRB counts in UR compared to UN as found in this study is marginal. As anticipated, the presence of the contaminant POS significantly reduces the number of THRB counts in both of the contaminated rhizosphere (CR) and non-rhizosphere (CN) treatments over the uncontaminated treatments (UN and UR). The microbial counts were much more pronounced in the uncontaminated soil than the contaminated soil because of the inhibitory effects of POS to the microorganism in general in the contaminated soil. However, there was a significant increase in the number of bacterial populations over time in the rhizosphere treatments (CR), as there were higher bacterial counts after 90 days of treatment compared to that of 30 days while a decrease in THRB counts was generally observed in CN. This shows that the presence of *C. cajan* favours the growth of THRB bacteria in the soil as found in other studies^22,27^. The result is also supported by a previous finding^28^, who reported a total decrease in the number of viable heterotrophic bacteria in soil contaminated with oily sludge.

**Figure 3:**
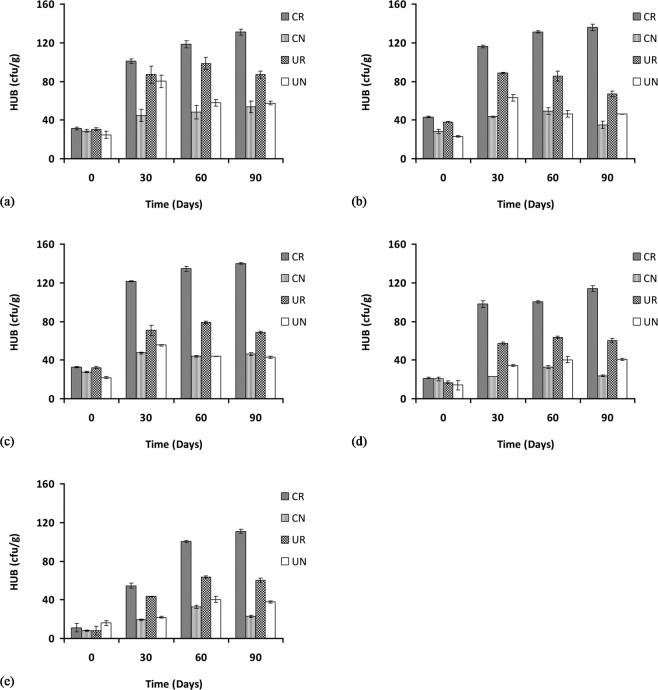
Hydrocarbon-utilizing rhizospheric bacterial counts at 1, 2, 3, 4, and 5% concentration of petroleum oily sludge labelled (**a**), (**b**), (**c**), (**d**) and (**e**), respectively, under various treatments presented as mean ± standard deviation (n = 3). CR: Contaminated rhizosphere, CN: Contaminated non-rhizosphere, UR: Uncontaminated rhizosphere, UN: Uncontaminated non-rhizosphere.

The hydrocarbon utilizing rhizosphere bacteria (HURB) at 1% POS from 0 to 90 days indicated a significant increase (p < 0.05) in bacterial count in all of the treatments such as in the contaminated rhizosphere CR (from 31.3 × 10^7^ to 131 × 10^7^ CFU/g), in the contaminated non-rhizosphere CN (from 28 × 10^7^ to 54 × 10^7^ CFU/g), uncontaminated rhizosphere UR (from 30 × 10^7^ to 86 × 10^7^ CFU/g) and in the uncontaminated non-rhizosphere UN (from 25 × 10^7^ to 58 × 10^7^ CFU/g) (Fig. 3(a)).

In the 2% POS concentration, the HURB counts from 0 to 90 days show a similar increase in counts where the contaminated rhizosphere CR bacterial counts increased significantly (p < 0.05) from 43 × 10^7^ to 136 × 10^7^ CFU/g, no significant increase (p > 0.05) in the contaminated non-rhizosphere CN (from 28 × 10^7^ to 35 × 10^7^ CFU/g), a significant increase (p < 0.05) for the bacterial count in the uncontaminated rhizosphere UR from 38 × 10^7^ to 67 × 10^7^ CFU/g), and a significant increase (p < 0.05) for the bacterial count in the uncontaminated non-rhizosphere UN as well (from 23 × 10^7^ to 46 × 10^7^ CFU/g) (Fig. 3(b)).

Similarly, for the 3% POS concentration, the HURB counts increased significantly (p < 0.05) from 0 to 90 days for all treatments such as in the contaminated rhizosphere CR (from 32 × 10^7^ to 140 × 10^7^ CFU/g), in the contaminated non-rhizosphere CN (from 28 × 10^7^ to 46 × 10^7^ CFU/g), in the uncontaminated rhizosphere UR (from 32 × 10^7^ to 67 × 10^7^ CFU/g) and finally in the uncontaminated non-rhizosphere UN (from 22 × 10^7^ to 43 × 10^7^ CFU/g) (Fig. 3(c)). For the 4% POS concentration, the counts for HURB for CR were significantly increased from 22 × 10^7^ to 115 × 10^7^ CFU/g, while no significant increase (p > 0.05) in the contaminated non-rhizosphere CN was observed from 20 × 10^7^ to 23 × 10^7^ CFU/g. A significant increase was observed for the uncontaminated rhizosphere UR from 17 × 10^7^ to 60 × 10^7^ CFU/g and also for the uncontaminated non-rhizosphere UN from 14 × 10^7^ to 41 × 10^7^ CFU/g (Fig. 3(d)). At the highest concentrations of POS tested (5%) HURB counts increased significantly (p < 0.05) for all treatments. The bacterial counts in the contaminated rhizosphere CR were increased from 11 × 10^7^ to 111 × 10^7^ CFU/g), while the contaminated non-rhizosphere CN counts were increased from 8 × 10^7^ to 22 × 10^7^ CFU/g. In the uncontaminated rhizosphere, the UR counts were increased from 8 × 10^7^ to 60 × 10^7^ CFU/g) and in the uncontaminated non-rhizosphere, the UN counts were increased from 16 × 10^7^ to 38.7 × 10^7^ CFU/g (Fig. 3(e)).

Hydrocarbon-utilizing rhizospheric bacterial counts revealed higher numbers in the contaminated rhizosphere (CR) for all of the POS concentrations tested, although the density tends to decrease as the POS concentration was increased. This result is similar to previous findings^29–32^, which include studies on the phytoremediation of soil amended with waste lubricating oil with *Jatropha curcaa*^31^ and *Hibiscus cannabinus*^29^ where significant increase in hydrocarbon-utilizing bacteria was observed after 30 days of incubation. However, both of these studies require the addition of organic wastes such as brewery spent grain (BSG) and spent mushroom compost (SMC) as additional carbon and nitrogen sources. The results obtained in this work demonstrate the stimulatory effect of rhizosphere to the hydrocarbon-utilizing bacterial population without the need for additional carbon or nitrogen sources.

Considering the complexity of the rhizosphere, a considerable number of microbial communities tend to withstand the toxic effect of the contaminant and are capable of using hydrocarbon as the source of carbon and energy compared to the community in the uncontaminated control. This is similarly reported in a study of epiphytic hydrocarbon-utilizing bacteria where the average number of bacterial density in a given contaminated soil is significantly greater than in the corresponding control, directly indicating that the contaminant is being utilized by the soil bacteria^33^. The results suggest that microbial enumeration is a direct indicative method to prove the response of microorganisms to hydrocarbons^34,35^. For a successful and effective phytoremediation process, the bacterial community in the hydrocarbon-contaminated soil must be well connected to the plant’s ability to enhance microbial association in the rhizosphere, resulting in a higher number of hydrocarbon-utilizing bacteria and enhancing their degradative capacity^36^.

Apart from the presence of petroleum oil which serves as the carbon and energy source, the higher population of hydrocarbon-degrading bacteria in the contaminated soil may also be attributed to the additive effect of the *C. cajan* roots which release organic compounds to further stimulate the degradation and bacterial growth. The increase in HURB counts in the presence of *C. cajans* observed in this study are also observed in several similar studies where higher counts of heterotrophic and oil-degrading bacteria were observed in contaminated rhizospheric soil than in the unplanted contaminated soil^8,37^.

### Culture-independent metagenomics analysis

**Figure 4:**
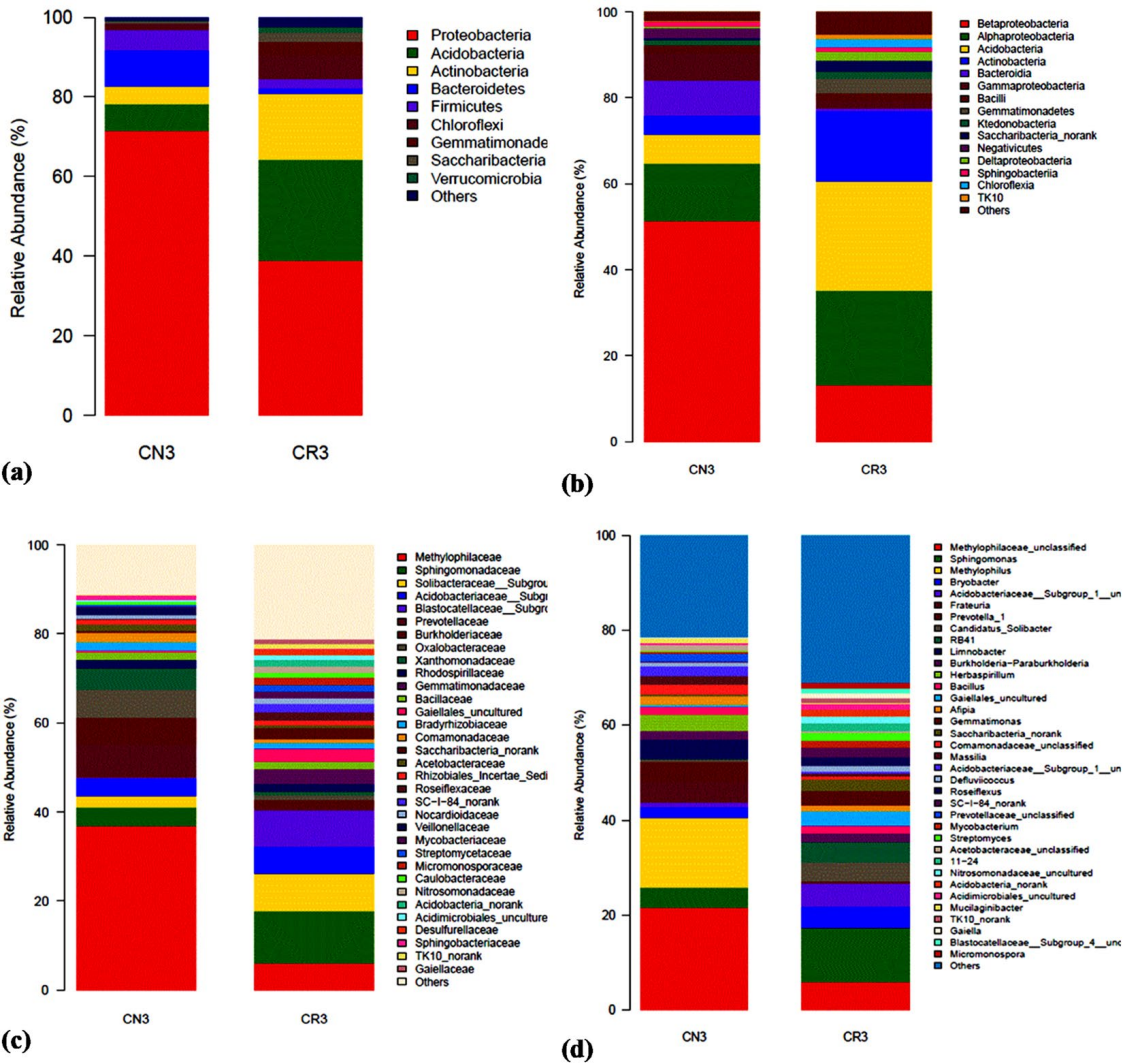
The relative abundance of bacterial taxa at the phylum (**a**), class (**b**), family (**c**) and genus (**d**) levels without (CN3) and with (CR3) *C. cajan* in soils contaminated with 3% petroleum oily sludge.

A sum of 59,873 and 59,756 sequences for CN3 and CR3 was found after a sequence optimization method, with an average number of 442.56 and 435.39 sequences for CN3 and CR3, respectively. A sum of 50821 and 47999 reads on CN3 and CR3, respectively, were subsampled from each replicate for further analysis. The calculated bacterial community abundance indices showed an increase in the values for Ace, Cho, Shannon (Shannon-Weaver) and the Simpson’s index in CR3 compared to CN3 (Table 1). In phylogeny, OTU is the most commonly applied microbial diversity unit where OTU is clustered with a cutoff of 97% similarity for the investigation of the abundance of group or species in the microbial community. The difference between CN3 and CR3 was seen in the number of OTU, which were 512 and 650 for CN3 and CR3, respectively, indicating an increase in richness upon the addition of *C. cajan*. A similar increase in OTU from 48 (control) to 62 (addition of rhizobacteria) is reported during the rhizoremediation of hexachlorobenzene in constructed wetlands^38^. The coverage indices showed a significant difference in the two treatments. The coverage did not change by much in this study. In general, a reduction in coverage rate indicates higher diversity. In a similar study of rhizoremediation of hexachlorobenzene using *T. angustifolia* rhizosphere and *P. australis* rhizosphere in constructed wetlands^38^, little reduction in the coverage index was observed for *T. angustifolia* rhizosphere treatment (from 50 to 47.5) while greater reduction in coverage was observed in *P. australis* rhizosphere soil treatment (from 50 to 29) despite both treatments showing nearly equal efficacy in remediating hexachlorobenzene. This may imply that coverage change alone may not be adequate in describing potential remediating ability of rhizodegraders. Of all the indices used in population diversity studies, the robust Shannon and Simpson indices have been recommended in measuring microbial diversity^39^, and it was observed that both of the Simpson’s (measured as InvSimpson) and the Shannon values for CR3 were higher than CN3. A change in both indicating an increase in diversity upon the introduction of *C. cajan* into the contaminated soil, which is a common theme seen in several studies involving phytoremediation using legumes^11,38,40^. Both measurements of population were lower in CN3, which may be due to the toxic effect of the contaminant on the bacterial community. The shift of soil bacterial community organization is also seen in the metagenomics sequences, and the results showed (Fig. 4a) that the contaminated rhizosphere (CR3) shows a diverse community of bacterial phyla, in comparison to the change of the microbial community structure seen in the contaminated non-rhizosphere (CN3). A lower diversity in CN3 may be the cause of a lower removal rate of petroleum hydrocarbon which has similarly been reported in previous studies^40,41^ where with an increase in the concentration of the contaminant, this results in an increase in the toxicity which reduces the efficiency of microbial degradation. This shows the important role of plant-like *C. cajan*, which stabilizes the C:N:P ratio for the effective degradation of hydrocarbon by the microbial community^22^. On the other hand, some study reported the inability of plant growth-promoting rhizobacteria to acquire nutrient for growth in severely polluted environments^41^.

**Table 1:** Richness and diversity of 16 s rRNA gene sequences from different treatments.

Samples	Reads	0.97					
		OTU	Ace	Chao	Coverage	Shannon	InvSimpson
CN3	50821	512	556	562	0.998	3.93	13.16
CR3	47999	650	656	660	0.999	5.33	66.67

Note: CN3 =  Contaminated non-rhizosphere with 3% oily sludge, CR3 =  Contaminated rhizosphere with 3% oily sludge.

**Figure 5:**
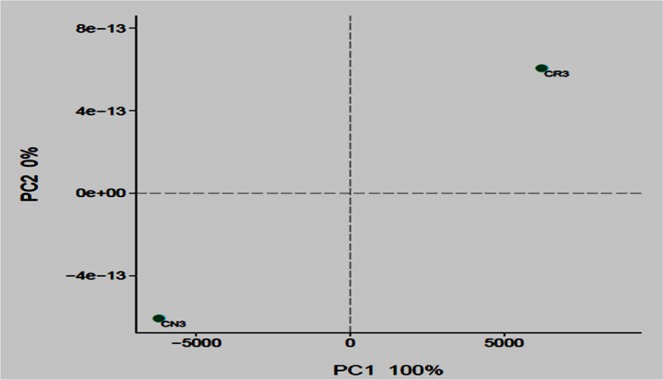
Bacterial 16 s rRNA community comparison of the different treatments analyzed using principle coordination analysis (PCoA). The proportions of variation by each ordination axis are indicated as percentages in parentheses. CN3 (contaminated non-rhizosphere), CR3 (contaminated rhizosphere).

The composition and taxonomic analysis of microbiota amplified sequences were categorized into eight phyla (CN3) and ten phyla in (CR3). The overall bacterial composition of the two treatments varied, as the distribution shows a similar variation between the two treatments in the phylum, class, family and genus level distributions, represented by Fig. 4a to d, respectively. This variation in microbial community is also shown by the PCoA plot, which revealed community-level differences between the contaminated non-rhizosphere control (CN3) and contaminated rhizosphere (CR3) microbiota (Fig. 5).

The contaminated non-rhizosphere (CN3) shows a trend of bacterial phylum such as *Proteobacteria, Actinobacteria, Acidobacteria, Bacteroidetes, Firmicutes, Chloroflexi, Saccharibacteria* and some uncategorized group. The contaminated rhizosphere (CR3) shows *Actinobacteria, Proteobacteria, Bacteroidetes, Acidobacteria, Firmicutes, Gemmatimonadetes, Saccharibacteria, Chloroflexi* and *Verrucomicrobia* to be the dominant phyla, and an unclassified group. In both of the treatments, the phylum *Proteobacteria* constitute the phylum with higher relative abundance accounting for > 60% in CN3 and almost 42% in CR3. In general, the phylum *Proteobacteria* dominated the communities of CN3 and CR3 at 70% and 42%, respectively. In the CN3, the *Proteobacteria* was dominated by the genus *Rhizobium, Sphingomonas*, and *Herbaspirillum*. The two phyla were not observed in CN3, but the phyla observed were *Verrucomicrobia and Gemmatimonadetes*. The presence of *Verrucomicrobia* is an indicator to the rhizospheric effect created by the presence of *C. cajan* as bacteria from this phylum are mostly found inhabiting grasslands and in subsurface soil horizons, where they were habitually the prevailing bacterial phylum^42^. Similarly, *Gemmatimonadetes* tend to dominant in soil with high rhizosphere activities, although their ecology remains poorly understood, and appear to be the dominant phyla in many soil bacterial communities; with bacteria from the phylum *Gemmatimonadetes* featuring nearly 2% of soil bacterial communities. Nevertheless, very little is understood of their ecology as a result of an insufficient study on the occurrence and ecology of this bacterial group^43^. The degradation of petroleum oily sludge hydrocarbons, in general, is accredited to indigenous microorganisms which are found in soil, but the presence of *C. cajan* will stimulate the habitat for the formation of favourable conditions of metabolisms to the microbial communities as demonstrated in this study where the culture-independent metagenomics technique to access a much more in-depth knowledge of the biological processes of petroleum oily sludge degradation during rhizodegradation shows promising results that agrees in principal to what was observed in the experiments.

An assessment at the phylum level identifies the bacteria belonging to the *Proteobacteria* as the richest community, and the richness was significantly increased. *Proteobacteria* covers a group of Gram-negative bacteria that have been widely reported to be able to degrade POS^41^.

### Biodegradation of petroleum oily sludge in soil

#### Gravimetric analysis

**Figure 6:**
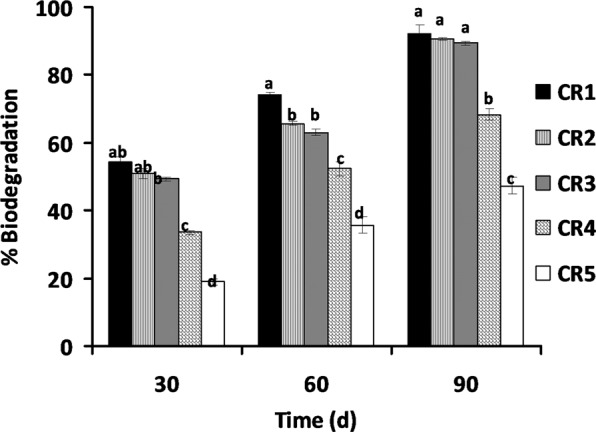
Percentage biodegradation of petroleum oily sludge contaminated soils by *C. cajan* under different treatments bars (means + SD, n = 3) with different letters within treatment days are significantly different based on LSD (p  < 0.05) CR1 to CR5: Contaminated rhizosphere 1 to 5% oil sludge, respectively.

The result of oily sludge degradation in the soil shows the effectiveness of *C. cajan* in plant-microbe bioremediation process. The result of the gravimetric analysis shows that almost 50% biodegradation of the oily sludge was observed at lower concentrations of oily sludge (CR1%, CR2% and CR3%) after 30 days of planting *C. cajan* (Fig. 6).

A low percentage of biodegradation (19%) was observed at the highest concentration of oily sludge tested (CR5%) after 30 days of planting with *C. cajan*. This may be as a result of inhibition at high concentrations of POS. The biodegradation shows significant (p < 0.05) increase after 60 days of planting of *C. cajan* for all oily sludge concentrations with CR1%, CR2%, CR3% and CR4% showing 74, 65, 63 and 52% biodegradation of POS, respectively, while CR5% was at 37% which also shows a significant increase albeit at a much lower percentage of degradation. A significant (p < 0.05) increase in biodegradation of POS was shown at the 90 days of the planting of *C. cajan* where CR1%, CR2%, CR3% and CR4% show 92, 90, 89 and 68% degradation, respectively. Likewise, CR5% had the lowest biodegradation at 47%. This result corresponds to the pattern of many studies observed in different plants, and the biodegradation also varies. In a previous study^31^, they reported that the phytoremediation of soil contaminated with 2.5 and 1% of spent engine oil using *J. curcas* at day 180 results in the 56.6% and 67.3% biodegradation of waste lubricating oil, respectively. Once the addition of organic waste to *J. curcas* was carried out, the remediation rapidly increases the removal of 2.5 and 1% spent engine oil by 89.6 and 96.6%, respectively. The variation is that the plant was stimulated with organic waste to achieve 96% biodegradation at 1% spent engine oil concentration whereas in this study, *C. cajan* being a legume plant, the addition of organic source is not necessary making *C. cajan* a better phytoremediating plant. Another study in the plant *Hibiscus cannabinus* for soil contaminated with 2.5 and 1% used lubricating oil for 90 days show the same pattern of biodegradation where the stimulation of the plant with organic waste resulted in the biodegradation of 86.4 and 91.8%, respectively, while in the unstimulated plant much lower biodegradations were observed at 52.5 and 58.9%, respectively, indicating the need for the addition of organic waste in non-nitrogen fixing plants^29^. Legumes have been shown to independently stimulate biodegradation of various forms of hydrocarbons including poly-aromatic hydrocarbons (PAHs) and their constituents^44^. In all of the phytoremediation studies, legumes are more effective at remediation than other non-legume plant species tested^15,17,20,44–50^. In some studies, the biodegradation of legume was reported to be lower at the beginning of the experiment but significantly increases as the experiments progresses^44^.

## Supplementary information

Datasets 1 to 3.

## Data Availability

All data generated or analyzed during this study are included in this published article (and its Supplementary Information files).
