# Supplementary material for: Rhizodegradation of Petroleum Oily Sludge-contaminated Soil Using Cajanus cajan Increases the Diversity of Soil Microbial Community
Source: Sci Rep. 2020 Mar 5;10:4094. doi: 10.1038/s41598-020-60668-1 (PMC7057954; doi:10.1038/s41598-020-60668-1)
Supplement: Supplementary file 1 — Datasets 1 to 3. [file 41598_2020_60668_MOESM1_ESM.docx]

**Rhizodegradation of Petroleum Oily Sludge-contaminated Soil Using *Cajanus cajan* Increases the Diversity of Soil Microbial Community**

Ibrahim Alkali Allamin ^1,2^, Mohd Izuan Effendi Halmi^3^, Nur Adeela Yasid^1^, Siti Aqlima Ahmad^1^ Siti Rozaimah Sheikh Abdullah^4^ and Yunus Shukor^1*^

^1^Department of Biochemistry, Faculty of Biotechnology and Biomolecular Sciences, Universiti Putra Malaysia, 43400 UPM Serdang, Selangor, Malaysia. Tel. 603-89466722. Fax +603-89430913. Email: [adeela@upm.edu.my](mailto:adeela@upm.edu.my) (Nur Adeela Yasid), [aqlima@upm.edu.my](mailto:aqlima@upm.edu.my) (Siti Aqlima Ahmad) [yunus.upm@gmail.com](mailto:yunus.upm@gmail.com) / [mohdyunus@upm.edu.my](mailto:mohdyunus@upm.edu.my) (Mohd Yunus Shukor)

^2^Department of Microbiology, Faculty of Sciences, University of Maiduguri, P.M.B. 1069, Maiduguri, Borno State, Nigeria. Ibrahim Alkali Allamin (email: [ibnallamin@gmail.com](mailto:ibnallamin@gmail.com) / [ibnallaminmcb@unimaid.edu.ng](mailto:ibnallaminmcb@unimaid.edu.ng)).

^3^Department of Land Management, Faculty of Agriculture, University Putra Malaysia, 43400 Serdang Selangor, Malaysia. Tel. +603-89474958/Fax: +603-8940 8316. Email: [adeela@upm.edu.my](mailto:adeela@upm.edu.my)

^4^Department of Chemical and Process Engineering, Faculty of Engineering and Built Environment, Universiti Kebangsaan Malaysia, UKM Bangi 43600, Selangor, Malaysia. Siti Rozaimah Sheikh Abdullah (email: [rozaimah@ukm.edu.my](mailto:rozaimah@ukm.edu.my))

Supporting information

Data for Figures

|   (a) |   (b) |
| --- | --- |
|   (c) |   (d) |
|   (e) |  |

**Data for Figure 2a.**

|  | CR | CN | UR | UN | stdev CR | stdev CN | stdev UR | stdev UN |
| --- | --- | --- | --- | --- | --- | --- | --- | --- |
| 0 | 125.8889 | 112 | 243.7778 | 202.5556 | 5.577734 | 3.774917 | 0.666667 | 15.13366 |
| 30 | 133.4444 | 93.33333 | 242.1111 | 167.8889 | 0.527046 | 4.527693 | 3.443996 | 0.600925 |
| 60 | 143.4444 | 88.44444 | 242.2222 | 157.8889 | 0.726483 | 0.527046 | 3.492054 | 1.166667 |
| 90 | 148.7778 | 77.33333 | 241.6667 | 157.5556 | 0.440959 | 0.707107 | 4.636809 | 0.726483 |

**Data for Figure 2b.**

|  | CR | CN | UR | UN | stdev CR | stdev CN | stdev UR | stdev UN |
| --- | --- | --- | --- | --- | --- | --- | --- | --- |
| 0 | 127.8889 | 66.33333 | 243.3333 | 221.7778 | 0.333333 | 0.5 | 0.5 | 0.440959 |
| 30 | 132.8889 | 55.11111 | 238.4444 | 198.8889 | 0.333333 | 0.333333 | 0.527046 | 3.333333 |
| 60 | 140 | 50.11111 | 222.3333 | 198.6667 | 0.5 | 0.333333 | 0.5 | 0.5 |
| 90 | 144.7778 | 44.77778 | 232 | 219.8889 | 0.440959 | 0.440959 | 11.51086 | 7.606649 |

**Data for Figure 2c.**

|  | CR | CN | UR | UN | stdev CR | stdev CN | stdev UR | stdev UN |
| --- | --- | --- | --- | --- | --- | --- | --- | --- |
| 0 | 123.5556 | 35.77778 | 152.5556 | 202.5556 | 12.17694 | 1.715938 | 7.875772 | 15.13366 |
| 30 | 122.1111 | 44.55556 | 244.3333 | 197.5556 | 0.333333 | 0.527046 | 0.5 | 2.877113 |
| 60 | 134.1111 | 34.11111 | 233.2222 | 176.5556 | 0.600925 | 1.269296 | 0.440959 | 0.527046 |
| 90 | 145.4444 | 31.33333 | 232 | 219.8889 | 0.527046 | 1.224745 | 11.51086 | 7.606649 |

**Data for Figure 2d.**

|  | CR | CN | UR | UN | stdev CR | stdev CN | stdev UR | stdev UN |
| --- | --- | --- | --- | --- | --- | --- | --- | --- |
| 0 | 96.66667 | 41.66667 | 233.3333 | 200.4444 | 3.278719 | 3.278719 | 0.5 | 0.881917 |
| 30 | 90.33333 | 27 | 222.4444 | 221.2222 | 0.5 | 3.807887 | 0.527046 | 16.67167 |
| 60 | 96.22222 | 32.66667 | 170 | 179.7778 | 0.440959 | 1.581139 | 5.43139 | 10.63929 |
| 90 | 112.1111 | 23.77778 | 232 | 219.8889 | 0.333333 | 0.666667 | 11.51086 | 7.606649 |

**Data for Figure 2e.**

|  | CR | CN | UR | UN | stdev CR | stdev CN | stdev UR | stdev UN |
| --- | --- | --- | --- | --- | --- | --- | --- | --- |
| 0 | 57.77778 | 23 | 243.3333 | 202.5556 | 0.440959 | 1.581139 | 0.5 | 15.13366 |
| 30 | 45.77778 | 19.22222 | 222.4444 | 198.5556 | 0.440959 | 0.833333 | 0.527046 | 0.527046 |
| 60 | 33.33333 | 14.33333 | 189.3333 | 188.1111 | 0.5 | 0.5 | 0.5 | 0.333333 |
| 90 | 45.44444 | 13.22222 | 223.5556 | 200.3333 | 0.726483 | 0.440959 | 3.94053 | 0.5 |

|   (a) |   (b) |
| --- | --- |
|   (c) |   (d) |
|   (e) |  |

**Data for Figure 3a.**

|  | CR | CN | UR | UN | stdev(CR) | stdev(CN) | stdev(UR) | stdev(UN) |
| --- | --- | --- | --- | --- | --- | --- | --- | --- |
| 0 | 31.33333 | 28.88889 | 30.66667 | 24.88889 | 1.581139 | 1.615893 | 1.581139 | 3.756476 |
| 30 | 101 | 44.88889 | 86.88889 | 80.11111 | 2.179449 | 6.333333 | 9.157571 | 6.735066 |
| 60 | 118.6667 | 48.11111 | 98.88889 | 57.88889 | 3.741657 | 6.808899 | 6.333333 | 3.443996 |
| 90 | 131.1111 | 53.77778 | 86.88889 | 57.55556 | 2.976762 | 6.13958 | 3.919325 | 1.878238 |

**Data for Figure 3b.**

|  | CR | CN | UR | UN | stdev(CR) | stdev(CN) | stdev(UR) | stdev(UN) |
| --- | --- | --- | --- | --- | --- | --- | --- | --- |
| 0 | 43.22222 | 28 | 38.11111 | 23 | 0.833333 | 2.12132 | 0.333333 | 0.866025 |
| 30 | 116.3333 | 43.44444 | 88.66667 | 63.11111 | 1.5 | 0.726483 | 0.707107 | 3.689324 |
| 60 | 131.1111 | 49.11111 | 85.44444 | 46.44444 | 1.166667 | 3.480102 | 5.246692 | 3.244654 |
| 90 | 136.1111 | 35.22222 | 67.11111 | 46.22222 | 3.480102 | 3.700601 | 2.848001 | 0.440959 |

**Data for Figure 3c.**

|  | CR | CN | UR | UN | stdev(CR) | stdev(CN) | stdev(UR) | stdev(UN) |
| --- | --- | --- | --- | --- | --- | --- | --- | --- |
| 0 | 32.66667 | 27.77778 | 32.33333 | 21.88889 | 21.88889 | 0.5 | 1.092906 | 1 |
| 30 | 121.6667 | 47.44444 | 70.88889 | 55.55556 | 55.55556 | 0.707107 | 0.881917 | 5.348936 |
| 60 | 134.6667 | 44 | 78.88889 | 43.88889 | 43.88889 | 2.645751 | 0.866025 | 1.452966 |
| 90 | 140 | 46.22222 | 68.55556 | 42.88889 | 42.88889 | 0.866025 | 1.563472 | 1.130388 |

**Data for Figure 3d.**

|  | CR | CN | UR | UN | stdev(CR) | stdev(CN) | stdev(UR) | stdev(UN) |
| --- | --- | --- | --- | --- | --- | --- | --- | --- |
| 0 | 21.44444 | 20.44444 | 16.88889 | 14.11111 | 0.881917 | 1.878238 | 1.452966 | 4.960959 |
| 30 | 97.88889 | 23 | 57.11111 | 34.22222 | 3.333333 | 0 | 1.452966 | 1.20185 |
| 60 | 100.1111 | 32.66667 | 63.66667 | 40.33333 | 1.166667 | 1.581139 | 1.224745 | 3.316625 |
| 90 | 114.1111 | 23.77778 | 60.22222 | 40.66667 | 2.848001 | 0.666667 | 2.108185 | 1 |

**Data for Figure 3e.**

|  | CR | CN | UR | UN | stdev(CR) | stdev(CN) | stdev(UR) | stdev(UN) |
| --- | --- | --- | --- | --- | --- | --- | --- | --- |
| 0 | 11.11111 | 8.222222 | 8 | 15.77778 | 4.371626 | 0.666667 | 4.609772 | 2.728451 |
| 30 | 54.55556 | 19.33333 | 43.33333 | 21.88889 | 2.603417 | 0.707107 | 0.5 | 0.781736 |
| 60 | 100.1111 | 32.66667 | 63.66667 | 40.33333 | 1.166667 | 1.581139 | 1.224745 | 3.316625 |
| 90 | 110.8889 | 22.88889 | 60.22222 | 37.88889 | 2.027588 | 1.054093 | 2.108185 | 1.054093 |

**Figure 6.** Percentage biodegradation of petroleum oily sludge contaminated soils by *C. cajan* under different treatments bars (means + SD, n=3) with different letters within treatment days are signiﬁcantly different based on LSD (p <0.05) CR1 to CR5: Contaminated rhizosphere 1 to 5% oil sludge, respectively.

|  | CR1 | CR2 | CR3 | CR4 | CR5 |
| --- | --- | --- | --- | --- | --- |
| 30 | 54.33333 | 51 | 49.33333 | 33.66667 | 19 |
| 60 | 74.33333 | 65.66667 | 63 | 52.33333 | 35.66667 |
| 90 | 92 | 90.66667 | 89.33333 | 68.33333 | 47.33333 |
